# The association between adipokines and pulmonary diseases: a mendelian randomization study

**DOI:** 10.1186/s12890-024-02863-8

**Published:** 2024-01-23

**Authors:** Dongcai Wu, Ziyuan Wang, Keju Wang, Yuhan Wang, Tan Wang

**Affiliations:** 1 College of Traditional Chinese Medicine, Changchun University of Chinese Medicine, Changchun, China; 2 Department of Respiratory, The Affiliated Hospital to Changchun University of Chinese Medicine, Changchun, China

**Keywords:** Adipokine, Adiponectin, Interstitial lung disease, Mendelian randomization, Causal relationships

## Abstract

**Background:**

The role of adipokines in the development of lung diseases is significant, yet their specific relationship with different lung diseases remains unclear.

**Methods:**

In our research, we analyzed genetic variations associated with adipokines and various lung conditions such as interstitial lung disease, chronic obstructive pulmonary disease, asthma, lung cancer, sleep apnea, pneumonia, and tuberculosis, using data from public genome-wide studies. We employed Mendelian randomization techniques, including inverse variance weighting, weighted median, and MR-Egger regression methods, and conducted sensitivity checks to validate our findings.

**Results:**

A study using the FinnGen database, which included 198,955 participants, identified 13 SNPs associated with adiponectin. Notably, adiponectin was found to significantly reduce the risk of interstitial lung disease and idiopathic pulmonary fibrosis. However, little evidence was found to establish a direct cause-effect relationship between the six adipokines and several other lung conditions, including sarcoidosis, asthma, chronic obstructive pulmonary disease, lung cancer, tuberculosis, pneumonia, and sleep apnea syndrome.

**Conclusion:**

This study reveals a reverse link between adiponectin levels and the likelihood of interstitial lung disease, including idiopathic pulmonary fibrosis.

**Supplementary Information:**

The online version contains supplementary material available at 10.1186/s12890-024-02863-8.

## Introduction

Adipokines are bioactive molecules synthesized within adipose tissue that exhibit hormone-like functionalities [1]. Aside from adiponectin and leptin, over 90% of all adipokines generated in adipose tissue derives from cellular sources other than adipocytes [2]. The systemic influences of adipokines primarily encompass the regulation of systemic inflammation through direct modulation of the intensity of immune response, degradation of the extracellular matrix, tissue responsiveness to additional endocrine stimuli, and cytokine production by immunocompetent cells [3].

Collectively, adipokines may play a crucial role in pulmonary diseases, particularly in the context of inflammatory disorders, malignancies, and interstitial lung diseases. However, the associations between adipokines and interstitial lung disease (ILD) [4], asthma [5], chronic obstructive pulmonary disease (COPD) [6], lung cancer [7], tuberculosis [8], sleep apnea syndrome (SAS) [9], and pneumonia [10] remain unclear. Causality cannot be deduced from the existing evidence.

Mendelian randomization (MR) analysis is an epidemiological method that uses genetic variation as instrumental variables (IVs) for exposure, effectively reducing residual confounding and minimizing reverse causality bias. This strengthens causal inference within exposure-outcome relationships [11]. Currently, MR analysis is widely used to investigate various associations, including identifying correlations between physiological markers and assessing the causal effects of diverse behaviors. Using existing genetic databases, genetic variants that influence adipokine levels can be considered as IVs to further examine the impact of adipokine levels on the risk of lung disease. Consequently, this study conducted MR analyses on two samples to elucidate the potential impact of genetically predicted levels of six adipokines [Adiponectin, Leptin, Soluble leptin receptor (sOB-R), Resistin, Retinol-binding protein 4 (RBP4), and plasminogen activator inhibitor 1 (PAI-1)] on lung disease.

## Materials and methods

### Study design

**Fig. 1:**
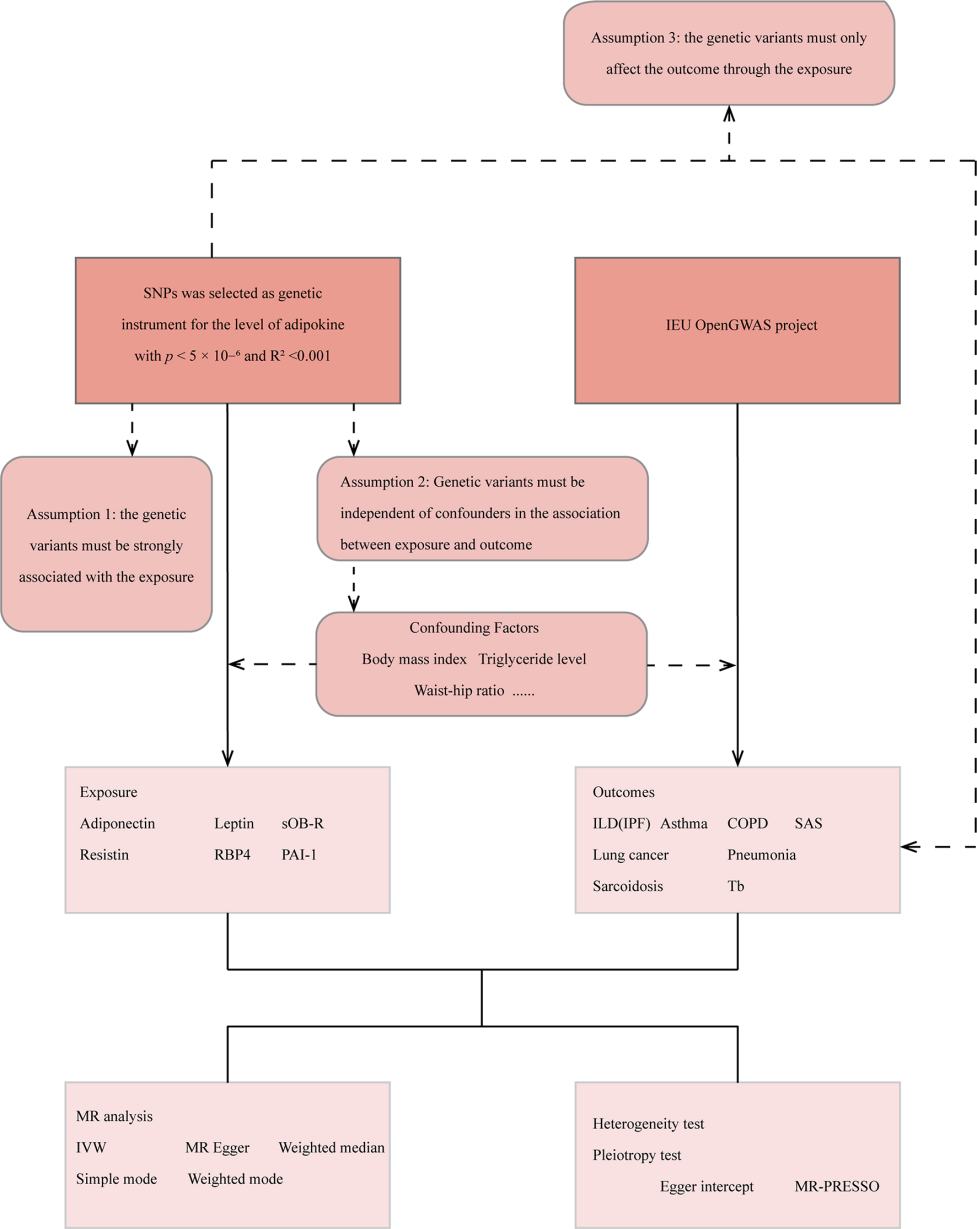
Shows the overall design of this study. MR = Mendelian randomization; IVW = inverse-variance weighted; MR-PRESSO = MR Pleiotropy RESidual Sum and Outlier; SNPs = single-nucleotide polymorphisms

A schematic representation of the study design is provided in Fig. 1. As the current study relied on previously published literature and publicly accessible databases, no additional ethical approval or participant consent was necessary.

### Data source

The data relevant to lung disease for this study were sourced from publicly accessible databases, specifically the IEU OpenGWAS project (https://gwas.mrcieu.ac.uk/), which consolidates various large-scale GWAS. This investigation focused on individuals of European ancestry, encompassing both males and females. The study included 198,955 participants of European ancestry, comprising 1,969 confirmed ILD cases and 196,986 controls, as well as IPF (GWAS ID: finn-b-IPF, *n* = 198,014). Additionally, Sarcoidosis (*n* = 486,673) and Sleep Apnea Syndrome (*n* = 476,853) were investigated through cross-population genetic association mapping of 220 human phenotypes at Osaka University [12]. Asthma (*n* = 462,933) and COPD (*n* = 462,933) data were obtained from the MRC Integrated Epidemiology Unit (MRC-IEU) consortium at the University of Bristol. Tuberculosis (*n* = 462,933) and pneumonia (*n* = 486,484) data were sourced from the UK Biobank. Lung cancer (*n* = 27,209) data came from the International Lung Cancer Consortium (ILCCO). Further details are provided in Supplementary Table.

### Selection of genetic variants for adipokines in IVs

Genetic variants associated with adiponectin levels in IVs were selected based on an extensive GWAS that included 39,883 individuals of European ancestry [13]. Additionally, a GWAS involving 32,161 individuals of European descent from 23 studies was used to identify IVs for leptin levels [14]. Since the summary-level data for the outcomes (Resistin, PAI-1, RBP4, sOB-R) were not available from the relevant GWAS, supplementary GWAS summary data for these four adipokines were obtained from the IEU OpenGWAS project. The corresponding GWAS IDs for Resistin, PAI-1, RBP4, and sOB-R are “prot-a-2524”, “prot-a-2696”, “prot-a-2507”, and “prot-a-1724”, respectively.

### Selection and validation of IVs

To investigate the causal association between a specific exposure and a particular outcome, three rigorous criteria must be met: (1) genetic variations should exhibit a strong correlation with the exposure; (2) these variations should be independent of confounders affecting the relationship between exposure and outcome; (3) these variations should influence the outcome only through the exposure pathway, eliminating pleiotropy and adhering to the exclusion restriction assumption [15]. Initially, SNPs were identified as having a strong association with the exposure if their *P* values were < 5 × 10 ^− 6^. Subsequently, SNPs exhibiting R^2^ > 0.001 and within 10,000 kb were deemed to possess substantial linkage disequilibrium (LD) and were consequently excluded from the analysis. Thirdly, the F-statistic was used to evaluate the strength of individual SNPs, with F-statistics surpassing 10 considered robust enough to alleviate potential bias [16]. Before conducting MR analyses, a data harmonization step was undertaken to ensure that SNP effects on exposure and outcome pertained to the same allele.

### Statistical analysis

The primary analysis involves applying an IVW approach based on a random effects model. The IVW method can be adjusted to approximate the variance of the inverses of these individual causal estimates [17]. Cochran’s Q statistic was computed to ascertain heterogeneity between results derived from separate SNPs, with *P* < 0.05 indicating significant heterogeneity. Both MR-Egger regression analysis and the MR-PRESSO global test were utilized to evaluate potential directional pleiotropy [18]. In conjunction with IVW, supplementary methods were employed to verify the consistency of the test outcomes. These included MR-Egger, weighted median, simple mode, and weighted mode approaches [19–20]. The MR-Egger method allows all SNPs to be invalid, while the weighted median method tolerates up to 50% of invalid SNPs. Odds ratios (*OR*) and 95% confidence intervals (95% CI) were employed to characterize the MR findings concerning the association between exposure and outcome. Furthermore, a leave-one-out sensitivity analysis was conducted to gauge the influence of individual SNPs on the overall estimation. Statistical significance is determined by a two-tailed *p*-value of less than 0.05. The analyses were performed using TwoSampleMR version 0.5.7 and MRPRESSO version 1.0, in R version 4.2.2.

## Results

### SNP selection

In this MR study, the selected SNPs for the six adipokine levels are presented in Supplementary Table. During the course of the current research, two SNPs were removed: rs6864862, which was associated with RBP4 levels, and rs10487505, which was related to leptin levels. Both were removed because they were palindromic with intermediate allele frequencies. As a result, the study utilized 15 SNPs as IVs for adiponectin levels, 4 SNPs for leptin levels, 16 SNPs for resistin levels, 14 SNPs for RBP4 levels, 11 SNPs for sOB-R levels, and 17 SNPs for PAI-1 levels. Notably, all the retained SNPs were located on distinct chromosomes, and therefore, none were removed due to linkage disequilibrium (LD) effects in the analysis. The F-statistics for the six adipokines exceeded 10, which confirmed the robustness of the instrumentation and reduced the likelihood of spurious associations driven by weak instrumental bias.

### Mendelian randomization analyses

**Fig. 2:**
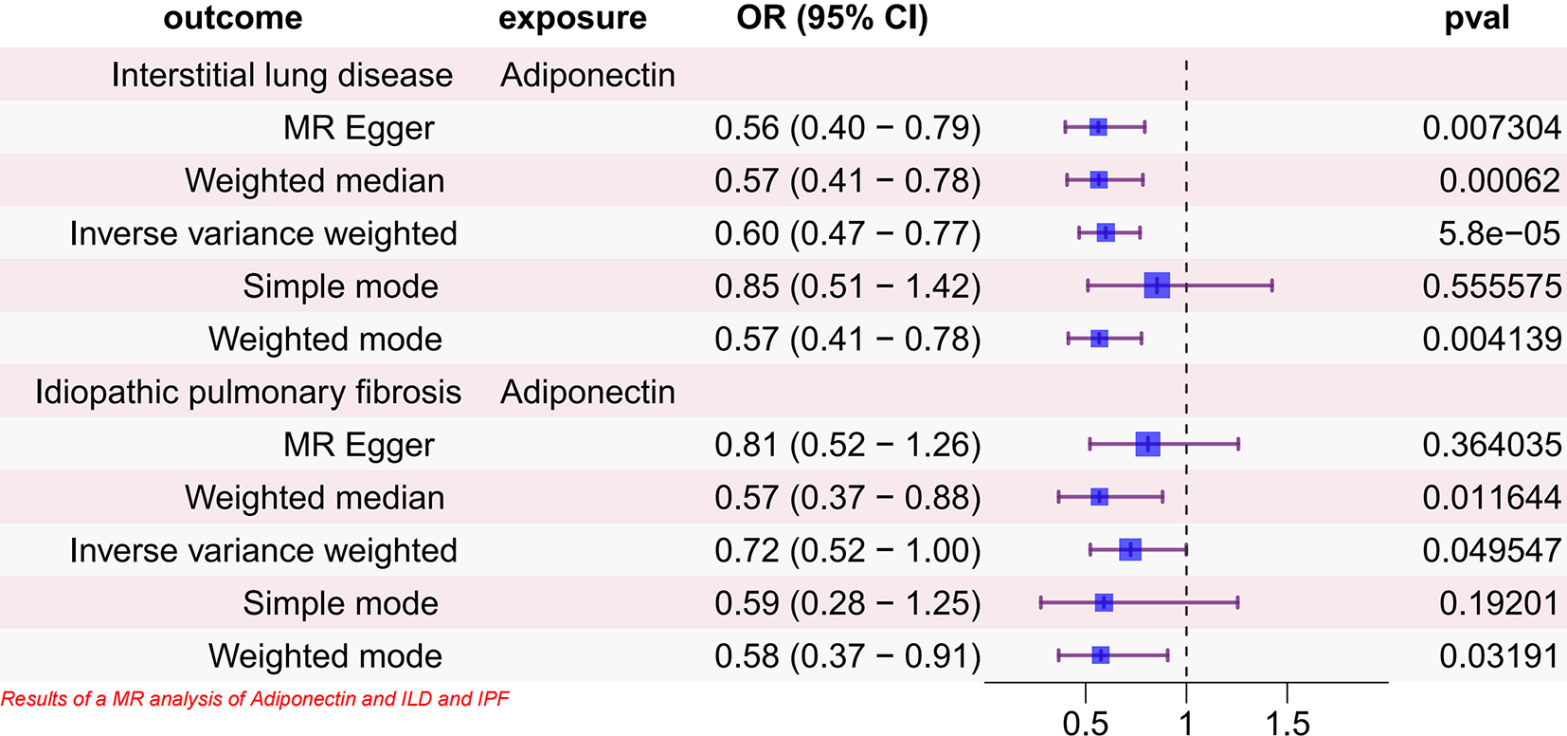
Forest plot of results of MR analysis of adiponectin and interstitial lung disease and idiopathic pulmonary fibrosis

The results showed that adiponectin, an adipokine, was negatively associated with the risk of ILD using the IVW method (*OR* = 0.59, 95% CI 0.46–0.76, *P* = 5.80 × 10 − 5). Other test methods produced the following results: MR-Egger (*OR* = 0.56, 95% CI = 0.40 ~ 0.79, *P* = 0.0073); Weighted median (*OR* = 0.56, 95% CI 0.40–0.78, *P* = 6.20 × 10 − 4); Simple mode (*OR* = 0.85, 95% CI 0.51–1.42, *P* = 0.556); Weighted mode (*OR* = 0.57, 95% CI 0.41–0.78, *P* = 0.0041). Furthermore, no evidence of horizontal pleiotropy was observed in the MR-Egger regression analysis (*P*-intercept = 0.605), and no strong heterogeneity was detected (*P* = 0.748). Additionally, scatterplots and leave-one-out plots in Supplementary Figures S1 corroborate the stability and dependability of the outcomes. It is worth mentioning that in the subgroup analysis, adiponectin was found to reduce the risk of IPF using the IVW method (*OR* = 0.72, 95% CI 0.52–0.99, *P* = 0.049). Other test methods showed the following results: MR-Egger (*OR* = 0.80, 95% CI = 0.52–1.26, *P* = 0.364); Weighted median (*OR* = 0.56, 95% CI 0.36–0.88, *P* = 0.012); Simple mode (*OR* = 0.59, 95% CI 0.28–1.25, *P* = 0.192); Weighted mode (*OR* = 0.58, 95% CI 0.37–0.91, *P* = 0.031). No evidence of horizontal pleiotropy was observed in the MR-Egger regression analysis (*P*-intercept = 0.605), and no strong heterogeneity was detected (*P* = 0.748). Furthermore, scatterplots and leave-one-out plots in Supplementary Figures S2 confirm the stability and reliability of the results. See Fig. 2 for details.

Regarding the causal relationship between adiponectin and sarcoidosis, asthma, COPD, lung cancer, tuberculosis, pneumonia, and sleep apnea syndrome, the IVW test results were as follows: (*OR* = 0.85, 95% CI 0.66–1.09, *P* = 0.208; *OR* = 0.99, 95% CI 0.98–1.01, *P* = 0.431; *OR* = 0.99, 95% CI 0.99–1.00, *P* = 0.998; *OR* = 0.91, 95% CI 0.75–1.11, *P* = 0.353; *OR* = 1.00, 95% CI 0.99–1.00, *P* = 0.607; *OR* = 0.97, 95% CI 0.86–1.10, *P* = 0.710; *OR* = 1.05, 95% CI 0.95–1.16, *P* = 0.352).

No significant causal relationships were found between leptin, sOB-R, resistin, RBP4, or PAI-1 and sarcoidosis, asthma, COPD, lung cancer, tuberculosis, pneumonia, and sleep apnea syndrome. Detailed analysis results can be found in Supplementary Table.

## Discussion

To the best of our knowledge, the present study is the first to employ Mendelian randomization to examine the relationship between adipokines and lung disease. The results obtained here reveal a statistically significant inverse correlation between adiponectin concentrations and the likelihood of developing interstitial lung disease.

Adiponectin (APN) is the most abundantly observed adipokine in peripheral blood circulation [21] and has been implicated in various biological processes, including lipid and insulin metabolism, apoptosis, and inflammatory response [22]. Primarily, adiponectin functions as a modulator of inflammation and fibrosis via the peroxisome proliferator-activated receptor-gamma (PPAR-γ) pathway [23]. A previous investigation reported that individuals with an elevated adiponectin-to-leptin ratio demonstrated poorer survival outcomes compared to those with severe IPF and a reduced adiponectin-to-leptin ratio [24]. Nonetheless, experimental research has demonstrated that adiponectin can suppress the expression of alpha-smooth muscle actin (α-SMA), type I collagen, and pro-inflammatory cytokines [25–26]. Adiponectin has been proposed as a potential therapeutic agent for fibrosis-associated pathologies, its antifibrotic properties potentially mediated through the inhibition of the nuclear factor-kappa B (NF-κB) signaling pathway [27]. Another study found that adiponectin mitigated paraquat-induced pulmonary fibrosis in a dose-responsive manner by inhibiting the activation of lung fibroblasts [28]. The adiponectin/carnitine palmitoyltransferase 1 A- (APN/CPT1A-) mediated fatty acid metabolism has been shown to confer partial protection against IPF through the activation of autophagy, offering novel avenues for IPF treatment [29]. As a result, the precise nature of the relationship between adiponectin and idiopathic pulmonary fibrosis remains a subject of ongoing debate. The current study clarifies a negative association between adiponectin levels and the risk of idiopathic pulmonary fibrosis from a genetic standpoint.

In adiponectin-deficient mice, alveolar macrophages undergo spontaneous activation, resulting in elevated release of tumor necrosis factor-alpha (TNF-α) and matrix metalloproteinase 12 (MMP-12) [30]. This process contributes to the structural distortion of the distal lung cavity [31], ultimately leading to the development of interstitial lung disease. Lower circulating adiponectin levels have been associated with severe subclinical lung inflammation, fibrosis, and reduced lung function [32]. Monitoring serum adiponectin levels during intravenous pulse cyclophosphamide (IVCY) treatment might assist in identifying systemic sclerosis patients with treatment-resistant interstitial lung disease and those at an increased risk for exacerbation during follow-up [33]. This study concluded that adiponectin mitigates the risk of interstitial lung disease.

Clinical research has demonstrated that pulmonary tuberculosis is characterized by diminished circulating adiponectin and leptin levels, as well as augmented resistin levels [8]. Serum adiponectin concentrations have been shown to provide protection against asthma in premenopausal women and adolescent girls [34]. Patients with COPD exhibit elevated systemic and airway leptin concentrations, indicative of increased airway inflammation and disease severity [35]. An observational study revealed that while leptin and adiponectin were not directly implicated in disease alterations in non-small cell lung carcinoma, resistin, functioning as a pro-inflammatory cytokine, might play a role in the pathogenesis of weight loss in NSCLC patients [36]. This study found limited evidence supporting a causal relationship between adiponectin, leptin, sOB-R, resistin, RBP4, or PAI-1 and sarcoidosis, asthma, COPD, lung cancer, tuberculosis, pneumonia, or sleep apnea syndrome.

There are several limitations to our study that need to be acknowledged. First, the statistical power of certain analyses may have been low due to a limited number of cases. This is particularly evident in the case of leptin levels, which could potentially lead to invalid results in some instances. Secondly, the data used in our study was sourced from publicly accessible GWAS summary-level datasets. However, the lack of detailed demographic information and clinical manifestations, such as age, sex, and disease progression, prevented us from conducting risk stratification and routine subgroup analyses. Lastly, the study population was confined to individuals of European descent. While this approach helps to minimize the impact of racial structural bias, it also implies that our findings may not be universally applicable to populations of other racial backgrounds.

### Electronic supplementary material

Below is the link to the electronic supplementary material.

**Supplementary Material 1: Supplementary Figures S1:** (A) Forest plots (B) leave-one-out plots (C) scatter plots for the outcome of Interstitial lung disease. **Supplementary Figures S2:** (A) Forest plots (B) leave-one-out plots (C) scatter plots for the outcome of Idiopathic pulmonary fibrosis

**Supplementary Material 2: Supplementary Table 1:** F-statistic for adipokines. **Supplementary Table 2:** Mendelian randomization (MR) results for adiponectin and lung disease. **Supplementary Table 3:** Mendelian randomization results for leptin and lung disease. **Supplementary Table 4:** Mendelian randomization results for leptin receptor and lung disease. **Supplementary Table 5:** Mendelian randomization results for retinol-binding protein 4 and lung disease. **Supplementary Table 6:** Mendelian randomization results for resistin and lung disease. **Supplementary Table 7:** Mendelian randomization results for plasminogen activator inhibitor 1 and lung disease

## Data Availability

During the execution of analytical procedures and the subsequent interpretation of findings, the research utilized open-access data resources, specifically the Genome-Wide Association Study (GWAS) data provided by the Integrative Epidemiology Unit (IEU) Open GWAS Project https://gwas.mrcieu.ac.uk/. The use of these datasets was essential for facilitating a comprehensive examination and understanding of the derived outcomes.

## Abbreviations

GWAS: genome-wide association study
IPF: Idiopathic pulmonary fibrosis
ILD: Interstitial lung disease
IEU: Integrative Epidemiology Unit
IVW: inverse-variance weighted
MR: Mendelian randomization
MR-PRESSO: MR Pleiotropy RESidual Sum and Outlier
sOB-R: Soluble leptin receptor
RBP4: Retinol-binding protein 4
PAI-1: Plasminogen activator inhibitor 1
