# Supplementary material for: The association between adipokines and pulmonary diseases: a mendelian randomization study
Source: BMC Pulm Med. 2024 Jan 23;24:50. doi: 10.1186/s12890-024-02863-8 (PMC10804699; doi:10.1186/s12890-024-02863-8)

Supplementary Figures S1: (A) Forest plots (B) leave-one-out plots (C) scatter plots for the outcome of Interstitial lung disease

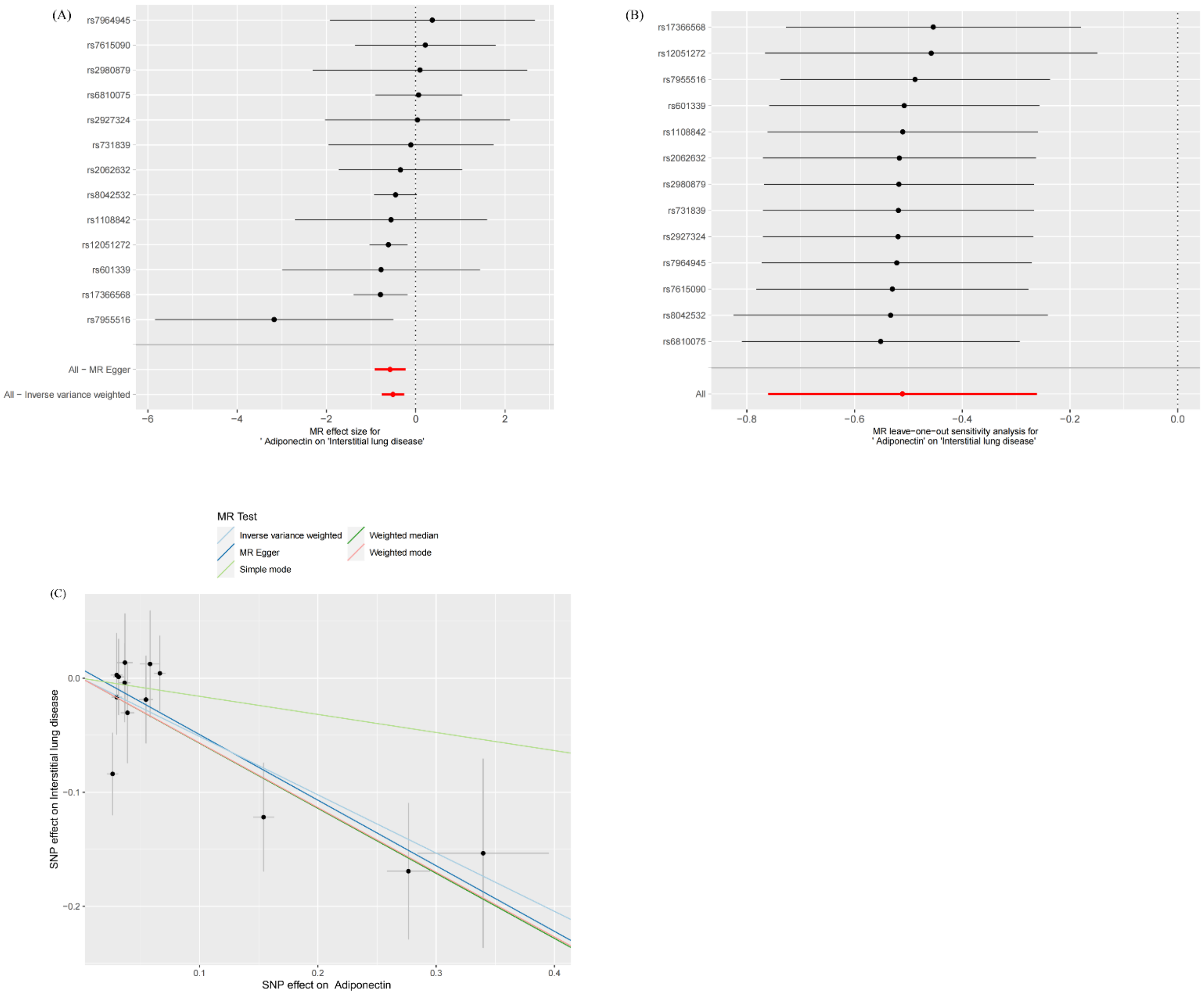

Supplementary Figures S2: (A) Forest plots (B) leave-one-out plots (C) scatter plots for the outcome of Idiopathic pulmonary fibrosis

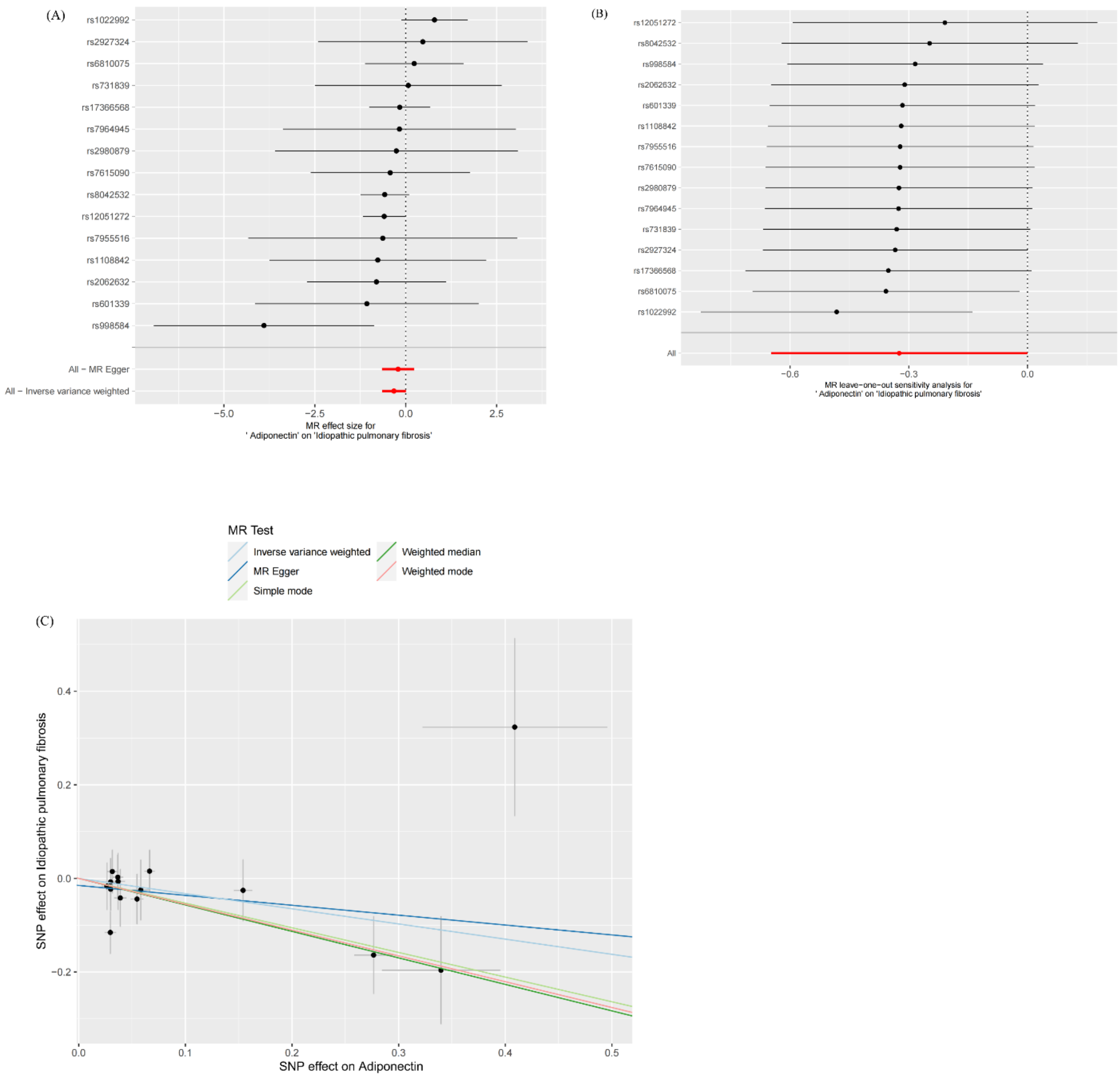

Supplement: Supplementary file 1 — Supplementary Material 1: Supplementary Figures S1: (A) Forest plots (B) leave-one-out plots (C) scatter plots for the outcome of Interstitial lung disease. Supplementary Figures S2: (A) Forest plots (B) leave-one-out plots (C) scatter plots for the outcome of Idiopathic pulmonary fibrosis [file 12890_2024_2863_MOESM1_ESM.pdf]
