# Supplementary material for: The association between adipokines and pulmonary diseases: a mendelian randomization study
Source: BMC Pulm Med. 2024 Jan 23;24:50. doi: 10.1186/s12890-024-02863-8 (PMC10804699; doi:10.1186/s12890-024-02863-8)
Supplement: Supplementary file 2 — Supplementary Material 2: Supplementary Table 1: F-statistic for adipokines. Supplementary Table 2: Mendelian randomization (MR) results for adiponectin and lung disease. Supplementary Table 3: Mendelian randomization results for leptin and lung disease. Supplementary Table 4: Mendelian randomization results for leptin receptor and lung disease. Supplementary Table 5: Mendelian randomization results for retinol-binding protein 4 and lung disease. Supplementary Table 6: Mendelian randomization results for resistin and lung disease. Supplementary Table 7: Mendelian randomization results for plasminogen activator inhibitor 1 and lung disease [file 12890_2024_2863_MOESM2_ESM.pdf]

Supplementary Material

Supplementary table 1: F-statistic for adipokines

Supplementary table 2: Mendelian randomization (MR) results for adiponectin and lung disease

Supplementary table 3: Mendelian randomization results for leptin and lung disease

Supplementary table 4: Mendelian randomization results for leptin receptor and lung disease

Supplementary table 5: Mendelian randomization results for retinol-binding protein 4 and lung disease

Supplementary table 6: Mendelian randomization results for resistin and lung disease

Supplementary table 7: Mendelian randomization results for plasmi nogen activator inhibitor 1 and lung disease

Supplementary table 1: F-statistics for adipokines, all greater than 10

Exposure	SNP	effect_allele.exposure	other_allele.exposure	chr.exposure	pos.exposure	beta.exposure	se.exposure	pval.exposure	F statistics
Adiponectin	rs17366568	A	G	3	186570453	-0.154104	0.008685	1.00E-200	116.4282
Adiponectin	rs2062632	C	T	3	186461181	-0.054666	0.005893	2.52E-19	37.77334
Adiponectin	rs7615090	G	T	3	186591003	-0.058149	0.008464	2.81E-11	20.45525
Adiponectin	rs6810075	C	T	3	186548565	-0.066402	0.004788	1.00E-200	60.17435
Adiponectin	rs1108842	C	A	3	52720080	0.029927	0.004382	3.66E-11	13.04568
Adiponectin	rs998584	A	C	6	43757896	-0.029627	0.005293	5.84E-08	12.84271
Adiponectin	rs2980879	T	A	8	126481475	0.029851	0.005059	1.08E-08	12.25318
Adiponectin	rs7955516	C	A	12	20498036	0.026448	0.004594	2.43E-08	10.11973
Adiponectin	rs7964945	A	T	12	124437668	0.036875	0.006419	2.61E-08	12.36176
Adiponectin	rs601339	G	A	12	123174743	0.039007	0.005719	3.87E-11	11.38157
Adiponectin	rs1022992	A	T	13	40869972	-0.408858	0.086509	4.66E-06	85.49091
Adiponectin	rs8042532	G	T	15	74255230	-0.339734	0.055421	2.86E-09	56.04191
Adiponectin	rs12051272	T	G	16	82663288	-0.276549	0.018107	1.00E-200	40.46009
Adiponectin	rs2927324	T	C	16	81512821	0.031514	0.00451	1.29E-11	14.53043
Adiponectin	rs731839	A	G	19	33899065	0.036626	0.004838	2.20E-13	17.33987
Leptin	rs6738627	A	G	2	165544450	0.0261	0.0057	4.57E-06	10.45642
Leptin	rs6973074	G	T	7	154485710	-0.0377	0.0081	3.05E-06	10.33518
Leptin	rs10487505	C	G	7	127860163	-0.0342	0.0051	2.70E-11	18.53323
Leptin	rs11597063	T	C	10	7477510	-0.0687	0.0149	3.85E-06	19.11958
Leptin	rs6071166	A	C	20	37333012	-0.0298	0.0054	2.86E-08	12.95086
Retinol-binding protein 4	rs143662949	C	T	3	10259317	0.6171	0.1195	2.40E-07	29.43649
Retinol-binding protein 4	rs4697146	T	C	4	20164914	-0.1221	0.026	2.51E-06	23.56288
Retinol-binding protein 4	rs115783813	A	C	5	49896349	-0.241	0.0499	1.38E-06	24.33923
Retinol-binding protein 4	rs13173873	A	G	5	178649283	-0.1323	0.0278	2.00E-06	23.18752
Retinol-binding protein 4	rs6864862	A	G	5	76159445	-0.1746	0.0333	1.55E-07	32.02655
Retinol-binding protein 4	rs498422	G	T	6	32286761	-0.2413	0.0525	4.17E-06	21.21693
Retinol-binding protein 4	rs112357560	A	G	10	72575542	-0.2332	0.0501	3.16E-06	22.38163
Retinol-binding protein 4	rs61864013	A	G	10	125169618	0.2779	0.0598	3.39E-06	24.07064
Retinol-binding protein 4	rs10882283	C	A	10	95360964	-0.1651	0.0273	1.38E-09	42.46656
Retinol-binding protein 4	rs148234495	A	G	12	33651484	0.6769	0.138	9.33E-07	31.31962
Retinol-binding protein 4	rs117597613	A	G	18	30947875	0.3629	0.0767	2.24E-06	24.70096
Retinol-binding protein 4	rs11084912	C	A	19	1859390	0.1523	0.0321	2.14E-06	22.34417
Retinol-binding protein 4	rs77691742	T	G	20	11924401	-0.1513	0.0328	3.89E-06	22.29383
Retinol-binding protein 4	rs8140446	A	T	22	49705242	0.2491	0.0512	1.12E-06	25.56938
Resistin	rs56402737	T	C	3	30656065	0.2757	0.0537	2.82E-07	31.50499
Resistin	rs7697212	T	C	4	25571317	0.3839	0.073	1.45E-07	32.01891
Resistin	rs2347113	G	A	5	151034828	-0.1164	0.0246	2.29E-06	22.48255
Resistin	rs149110519	T	C	6	144385777	0.4171	0.0644	9.33E-11	44.23019
Resistin	rs17073300	G	A	6	144349648	-0.2334	0.0404	7.41E-09	34.57239
Resistin	rs146013471	G	A	7	85048232	0.4561	0.0983	3.47E-06	26.93589
Resistin	rs4368939	T	A	8	11537295	0.1305	0.0285	4.57E-06	22.73913
Resistin	rs78651608	C	A	8	55484174	0.2463	0.0537	4.57E-06	21.91764
Resistin	rs117840977	A	G	8	73946664	-0.2507	0.0529	2.19E-06	24.67346
Resistin	rs4305862	C	T	8	130032394	-0.3169	0.0636	6.31E-07	25.98845
Resistin	rs3132809	C	G	11	134146406	-0.1246	0.0255	9.77E-07	24.97775
Resistin	rs11040140	T	C	11	48944728	-0.1335	0.0282	2.24E-06	29.63486
Resistin	rs60666652	C	T	17	38148272	0.1419	0.026	4.90E-08	31.34872
Resistin	rs10424785	C	T	19	45295032	0.3229	0.0698	3.80E-06	23.12723
Resistin	rs34124816	C	A	19	7733676	-0.6083	0.0645	4.37E-21	90.84946
Resistin	rs1423096	C	T	19	7739177	-0.1934	0.0411	2.57E-06	23.42731
Leptin receptor	rs78007650	A	G	1	65701309	-0.3908	0.0594	4.79E-11	49.39983
Leptin receptor	rs11208731	A	G	1	66219586	0.2457	0.0446	3.55E-08	33.28361
Leptin receptor	rs72644155	G	A	1	200118332	0.582	0.1168	6.17E-07	26.40117
Leptin receptor	rs10493380	C	A	1	66046117	-1.0473	0.0262	1.00E-200	1689.71
Leptin receptor	rs143084794	T	A	2	215680987	0.6078	0.1301	3.02E-06	25.49605
Leptin receptor	rs1933739	C	T	6	116309044	0.1289	0.0254	4.07E-07	24.38041
Leptin receptor	rs1998260	A	G	6	123893038	-0.1372	0.0273	4.90E-07	28.01177
Leptin receptor	rs1542465	C	T	11	81509575	-0.1332	0.0281	2.09E-06	23.97251
Leptin receptor	rs76598363	C	T	13	99015137	-0.2396	0.051	2.63E-06	21.80938
Leptin receptor	rs143351193	A	T	15	66063049	-0.5404	0.1129	1.70E-06	26.69991
Leptin receptor	rs497308	G	A	18	6029122	-0.2467	0.0529	3.09E-06	23.32327
Plasminogen activator inhibitor 1	rs1473794	C	A	1	34057453	-0.1434	0.0314	4.90E-06	22.91047
Plasminogen activator inhibitor 1	rs12566888	T	G	1	156869047	-0.1998	0.0419	1.82E-06	23.51059
Plasminogen activator inhibitor 1	rs150104310	T	A	1	218161147	0.3691	0.0801	4.07E-06	23.5258
Plasminogen activator inhibitor 1	rs116760026	G	C	2	140991825	0.2807	0.0614	4.79E-06	21.99463
Plasminogen activator inhibitor 1	rs17223780	T	C	3	42363369	0.1245	0.0271	4.47E-06	23.8075
Plasminogen activator inhibitor 1	rs2237053	C	T	4	110911080	-0.1228	0.0256	1.70E-06	23.48229
Plasminogen activator inhibitor 1	rs73009097	C	T	6	146722456	0.3696	0.08	3.80E-06	22.03872
Plasminogen activator inhibitor 1	rs114067707	A	G	6	160901411	0.2095	0.0445	2.51E-06	22.86012
Plasminogen activator inhibitor 1	rs6993770	T	A	8	106581528	-0.202	0.0273	1.38E-13	55.45775
Plasminogen activator inhibitor 1	rs78497424	T	G	9	109343818	0.4181	0.0876	1.78E-06	23.12138
Plasminogen activator inhibitor 1	rs2597576	T	C	11	97768593	0.1206	0.0263	4.37E-06	22.31142
Plasminogen activator inhibitor 1	rs75708520	C	T	12	91140078	0.3193	0.0683	2.88E-06	24.85727
Plasminogen activator inhibitor 1	rs117117424	C	T	12	128785456	0.4669	0.1021	4.79E-06	21.33265
Plasminogen activator inhibitor 1	rs1479485	G	C	12	5274659	-0.1728	0.0356	1.23E-06	23.87371
Plasminogen activator inhibitor 1	rs2745108	C	T	16	1547477	-0.1856	0.0402	3.80E-06	22.94998
Plasminogen activator inhibitor 1	rs9902160	A	G	17	76504331	0.1431	0.0308	3.47E-06	21.80225
Plasminogen activator inhibitor 1	rs73036903	C	G	19	48301989	-0.1931	0.0421	4.57E-06	21.30452

Supplementary table 2: The study used MR to estimate the relationship between adiponectin and lung disease.

id.exposure	exposure	id.outcome	outcome	method	nsnp	b	se	pval	OR	95%CI lower	95%CI upper
ieu-a-1	Adiponectin	finn-b-ILD	Interstitial lung disease	MR Egger	13	-0.575189	0.17524	0.007304	0.562598	0.399053773	0.79316886
ieu-a-1	Adiponectin	finn-b-ILD	Interstitial lung disease	Weighted median	13	-0.570441	0.166672	0.00062	0.565276	0.407743231	0.783671981
ieu-a-1	Adiponectin	finn-b-ILD	Interstitial lung disease	Inverse variance weighted	13	-0.511138	0.127119	5.80E-05	0.599813	0.467530306	0.769522997
ieu-a-1	Adiponectin	finn-b-ILD	Interstitial lung disease	Simple mode	13	-0.15861	0.261578	0.555575	0.853329	0.511042626	1.424873512
ieu-a-1	Adiponectin	finn-b-ILD	Interstitial lung disease	Weighted mode	13	-0.567154	0.160625	0.004139	0.567137	0.413963212	0.77698891
ieu-a-1	Adiponectin	finn-b-IPF	Idiopathic pulmonary fibrosis	MR Egger	15	-0.211356	0.224691	0.364035	0.809486	0.521132986	1.257390336
ieu-a-1	Adiponectin	finn-b-IPF	Idiopathic pulmonary fibrosis	Weighted median	15	-0.566043	0.224375	0.011644	0.567768	0.365745657	0.881378678
ieu-a-1	Adiponectin	finn-b-IPF	Idiopathic pulmonary fibrosis	Inverse variance weighted	15	-0.324314	0.165142	0.049547	0.723023	0.523094921	0.99936413
ieu-a-1	Adiponectin	finn-b-IPF	Idiopathic pulmonary fibrosis	Simple mode	15	-0.527118	0.384529	0.19201	0.590304	0.277816748	1.254275958
ieu-a-1	Adiponectin	finn-b-IPF	Idiopathic pulmonary fibrosis	Weighted mode	15	-0.551707	0.23155	0.03191	0.575966	0.365844993	0.906767478
ieu-a-1	Adiponectin	ukb-b-18113	Asthma	MR Egger	15	-0.001301	0.006191	0.836836	0.9987	0.986655331	1.010891892
ieu-a-1	Adiponectin	ukb-b-18113	Asthma	Weighted median	15	-0.003915	0.004233	0.355023	0.996093	0.987863235	1.004390969
ieu-a-1	Adiponectin	ukb-b-18113	Asthma	Inverse variance weighted	15	-0.003332	0.004228	0.4306	0.996673	0.988447623	1.004966979
ieu-a-1	Adiponectin	ukb-b-18113	Asthma	Simple mode	15	-0.006301	0.007233	0.398363	0.993718	0.979729698	1.007907034
ieu-a-1	Adiponectin	ukb-b-18113	Asthma	Weighted mode	15	-0.002077	0.004092	0.619667	0.997925	0.989953229	1.005961309
ieu-a-1	Adiponectin	ebi-a-GCST90018918	Sarcoidosis	MR Egger	15	-0.291299	0.168419	0.107355	0.747292	0.537191649	1.039564472
ieu-a-1	Adiponectin	ebi-a-GCST90018918	Sarcoidosis	Weighted median	15	-0.112765	0.17905	0.528828	0.893361	0.628950697	1.268928272
ieu-a-1	Adiponectin	ebi-a-GCST90018918	Sarcoidosis	Inverse variance weighted	15	-0.157588	0.125263	0.208368	0.854201	0.66824338	1.091907548
ieu-a-1	Adiponectin	ebi-a-GCST90018918	Sarcoidosis	Simple mode	15	-0.253236	0.27374	0.370588	0.776285	0.453950932	1.327495892
ieu-a-1	Adiponectin	ebi-a-GCST90018918	Sarcoidosis	Weighted mode	15	-0.086719	0.198929	0.669535	0.916935	0.620878792	1.35416047
ieu-a-1	Adiponectin	ukb-b-13447	Chronic obstructive pulmonary disease	MR Egger	8	0.00306	0.004377	0.510677	1.003065	0.994495642	1.011707921
ieu-a-1	Adiponectin	ukb-b-13447	Chronic obstructive pulmonary disease	Weighted median	8	0.00089	0.001573	0.571381	1.000891	0.997809632	1.0039814
ieu-a-1	Adiponectin	ukb-b-13447	Chronic obstructive pulmonary disease	Inverse variance weighted	8	-3.11E-06	0.001417	0.998247	0.999997	0.997223427	1.002778062
ieu-a-1	Adiponectin	ukb-b-13447	Chronic obstructive pulmonary disease	Simple mode	8	0.002256	0.003	0.476522	1.002258	0.996383378	1.008168166
ieu-a-1	Adiponectin	ukb-b-13447	Chronic obstructive pulmonary disease	Weighted mode	8	0.002422	0.001997	0.264552	1.002424	0.99850914	1.00635513
ieu-a-1	Adiponectin	ieu-b-4976	Pneumonia	MR Egger	15	-0.056827	0.088911	0.533831	0.944758	0.793667075	1.124612111
ieu-a-1	Adiponectin	ieu-b-4976	Pneumonia	Weighted median	15	-0.022699	0.067767	0.737657	0.977557	0.855967968	1.116416651
ieu-a-1	Adiponectin	ieu-b-4976	Pneumonia	Inverse variance weighted	15	-0.022456	0.060486	0.710447	0.977794	0.868481957	1.100865646
ieu-a-1	Adiponectin	ieu-b-4976	Pneumonia	Simple mode	15	-0.061122	0.095739	0.533505	0.940709	0.779759254	1.134879796
ieu-a-1	Adiponectin	ieu-b-4976	Pneumonia	Weighted mode	15	-0.042557	0.059799	0.488358	0.958336	0.85234604	1.077505381
ieu-a-1	Adiponectin	ieu-a-966	Lung cancer	MR Egger	14	-0.177642	0.147187	0.250717	0.837242	0.627426988	1.117220037
ieu-a-1	Adiponectin	ieu-a-966	Lung cancer	Weighted median	14	-0.230347	0.132647	0.082468	0.794258	0.612421099	1.030084736
ieu-a-1	Adiponectin	ieu-a-966	Lung cancer	Inverse variance weighted	14	-0.091783	0.098895	0.353362	0.912303	0.751550527	1.107439553
ieu-a-1	Adiponectin	ieu-a-966	Lung cancer	Simple mode	14	0.217445	0.198479	0.293155	1.242897	0.842338062	1.833934038
ieu-a-1	Adiponectin	ieu-a-966	Lung cancer	Weighted mode	14	-0.177568	0.124734	0.178128	0.837304	0.655703412	1.0692009
ieu-a-1	Adiponectin	ebi-a-GCST90018916	Sleep apnea syndrome	MR Egger	15	0.126912	0.068199	0.085519	1.135317	0.99326557	1.297683979
ieu-a-1	Adiponectin	ebi-a-GCST90018916	Sleep apnea syndrome	Weighted median	15	0.082811	0.063166	0.189859	1.086336	0.959833874	1.229510855
ieu-a-1	Adiponectin	ebi-a-GCST90018916	Sleep apnea syndrome	Inverse variance weighted	15	0.046798	0.050343	0.35259	1.04791	0.949447666	1.15658419
ieu-a-1	Adiponectin	ebi-a-GCST90018916	Sleep apnea syndrome	Simple mode	15	0.033485	0.08969	0.714488	1.034052	0.867354583	1.232786608
ieu-a-1	Adiponectin	ebi-a-GCST90018916	Sleep apnea syndrome	Weighted mode	15	0.075867	0.064411	0.25849	1.078819	0.950868986	1.223986488
ieu-a-1	Adiponectin	ukb-b-15622	Tuberculosis (Tb)	MR Egger	9	0.002934	0.004443	0.530148	1.002938	0.994242958	1.011708969
ieu-a-1	Adiponectin	ukb-b-15622	Tuberculosis (Tb)	Weighted median	9	0.000391	0.001729	0.821284	1.000391	0.997005703	1.003787216
ieu-a-1	Adiponectin	ukb-b-15622	Tuberculosis (Tb)	Inverse variance weighted	9	0.000702	0.001367	0.60767	1.000702	0.99802463	1.003386561
ieu-a-1	Adiponectin	ukb-b-15622	Tuberculosis (Tb)	Simple mode	9	0.000787	0.0027	0.777975	1.000788	0.995505547	1.006098011
ieu-a-1	Adiponectin	ukb-b-15622	Tuberculosis (Tb)	Weighted mode	9	0.000309	0.00216	0.889718	1.000309	0.996083187	1.004553224

Supplementary table 3: The study used MR to estimate the relationship between leptin and lung disease.

id.exposure	exposure	id.outcome	outcome	method	nsnp	b	se	pval	OR	95%CI lower	95%CI upper
ieu-a-1002	Leptin	finn-b-ILD	Interstitial lung disease	MR Egger	4	-1.853109	2.517795	0.538344	0.156749	0.001127235	21.79692709
ieu-a-1002	Leptin	finn-b-ILD	Interstitial lung disease	Weighted median	4	-0.58356	0.790737	0.460517	0.557909	0.118433483	2.628158288
ieu-a-1002	Leptin	finn-b-ILD	Interstitial lung disease	Inverse variance weighted	4	-0.556889	0.657995	0.397362	0.572989	0.157779092	2.080861148
ieu-a-1002	Leptin	finn-b-ILD	Interstitial lung disease	Simple mode	4	-0.567778	1.0991	0.641113	0.566783	0.06574216	4.886412679
ieu-a-1002	Leptin	finn-b-ILD	Interstitial lung disease	Weighted mode	4	-0.602244	1.04956	0.606294	0.547581	0.069991482	4.284026824
ieu-a-1002	Leptin	finn-b-IPF	Idiopathic pulmonary fibrosis	MR Egger	4	-6.228058	3.507378	0.217769	0.001973	2.04E-06	1.908661997
ieu-a-1002	Leptin	finn-b-IPF	Idiopathic pulmonary fibrosis	Weighted median	4	-1.070017	1.152157	0.353041	0.343003	0.035855997	3.281203995
ieu-a-1002	Leptin	finn-b-IPF	Idiopathic pulmonary fibrosis	Inverse variance weighted	4	-0.82549	0.971951	0.395708	0.43802	0.065185783	2.943307577
ieu-a-1002	Leptin	finn-b-IPF	Idiopathic pulmonary fibrosis	Simple mode	4	-2.333305	1.790662	0.283554	0.096975	0.002900123	3.242651785
ieu-a-1002	Leptin	finn-b-IPF	Idiopathic pulmonary fibrosis	Weighted mode	4	-1.788403	1.557648	0.33416	0.167227	0.00789605	3.541630801
ieu-a-1002	Leptin	ukb-b-18113	Asthma	MR Egger	4	0.036257	0.039383	0.454433	1.036923	0.959892599	1.120134272
ieu-a-1002	Leptin	ukb-b-18113	Asthma	Weighted median	4	-0.002531	0.014369	0.860205	0.997473	0.969771994	1.025964413
ieu-a-1002	Leptin	ukb-b-18113	Asthma	Inverse variance weighted	4	0.001708	0.012419	0.890602	1.00171	0.977621187	1.026391545
ieu-a-1002	Leptin	ukb-b-18113	Asthma	Simple mode	4	-0.004212	0.020739	0.852048	0.995797	0.956130681	1.037108211
ieu-a-1002	Leptin	ukb-b-18113	Asthma	Weighted mode	4	-0.004212	0.020797	0.852452	0.995797	0.956022361	1.037225718
ieu-a-1002	Leptin	ebi-a-GCST90018918	Sarcoidosis	MR Egger	4	1.296625	3.035008	0.710815	3.656932	0.009542586	1401.418174
ieu-a-1002	Leptin	ebi-a-GCST90018918	Sarcoidosis	Weighted median	4	-0.470732	0.81301	0.562589	0.624545	0.126915832	3.073344102
ieu-a-1002	Leptin	ebi-a-GCST90018918	Sarcoidosis	Inverse variance weighted	4	-0.591114	0.749627	0.430378	0.55371	0.127405111	2.406454756
ieu-a-1002	Leptin	ebi-a-GCST90018918	Sarcoidosis	Simple mode	4	-1.829918	1.418643	0.28752	0.160427	0.009947266	2.587317688
ieu-a-1002	Leptin	ebi-a-GCST90018918	Sarcoidosis	Weighted mode	4	0.497175	1.20565	0.707765	1.644071	0.154756993	17.46588666
ieu-a-1002	Leptin	ukb-b-13447	Chronic obstructive pulmonary disease	MR Egger	NA	NA	NA	NA	NA	NA	NA
ieu-a-1002	Leptin	ukb-b-13447	Chronic obstructive pulmonary disease	Weighted median	NA	NA	NA	NA	NA	NA	NA
ieu-a-1002	Leptin	ukb-b-13447	Chronic obstructive pulmonary disease	Inverse variance weighted	2	0.001947	0.003236	0.54744	1.001949	0.995613855	1.008323819
ieu-a-1002	Leptin	ukb-b-13447	Chronic obstructive pulmonary disease	Simple mode	NA	NA	NA	NA	NA	NA	NA
ieu-a-1002	Leptin	ukb-b-13447	Chronic obstructive pulmonary disease	Weighted mode	NA	NA	NA	NA	NA	NA	NA
ieu-a-1002	Leptin	ieu-b-4976	Pneumonia	MR Egger	4	0.148469	0.591842	0.825342	1.160057	0.363658277	3.700541288
ieu-a-1002	Leptin	ieu-b-4976	Pneumonia	Weighted median	4	0.177591	0.215249	0.409342	1.194337	0.783255751	1.821168171
ieu-a-1002	Leptin	ieu-b-4976	Pneumonia	Inverse variance weighted	4	0.207264	0.186165	0.265563	1.230308	0.854175638	1.772067515
ieu-a-1002	Leptin	ieu-b-4976	Pneumonia	Simple mode	4	0.073977	0.315633	0.829777	1.076783	0.580036963	1.998942674
ieu-a-1002	Leptin	ieu-b-4976	Pneumonia	Weighted mode	4	0.084874	0.282791	0.783672	1.08858	0.625380439	1.89485824
ieu-a-1002	Leptin	ieu-a-966	Lung cancer	MR Egger	4	-0.428684	1.062237	0.72559	0.651366	0.08121395	5.224197691
ieu-a-1002	Leptin	ieu-a-966	Lung cancer	Weighted median	4	-0.397876	0.39484	0.313604	0.671746	0.309820425	1.456463379
ieu-a-1002	Leptin	ieu-a-966	Lung cancer	Inverse variance weighted	4	-0.384675	0.334662	0.250374	0.680672	0.353237946	1.311620679
ieu-a-1002	Leptin	ieu-a-966	Lung cancer	Simple mode	4	-0.437374	0.508907	0.453293	0.64573	0.238155369	1.750818955
ieu-a-1002	Leptin	ieu-a-966	Lung cancer	Weighted mode	4	-0.451482	0.511808	0.442664	0.636684	0.233487808	1.736136415
ieu-a-1002	Leptin	ebi-a-GCST90018916	Sleep apnea syndrome	MR Egger	4	-0.531771	0.896555	0.613235	0.587564	0.101365806	3.405792429
ieu-a-1002	Leptin	ebi-a-GCST90018916	Sleep apnea syndrome	Weighted median	4	-0.182924	0.285993	0.522426	0.832831	0.475460978	1.458811757
ieu-a-1002	Leptin	ebi-a-GCST90018916	Sleep apnea syndrome	Inverse variance weighted	4	-0.201733	0.253079	0.425385	0.817313	0.497694974	1.342189295
ieu-a-1002	Leptin	ebi-a-GCST90018916	Sleep apnea syndrome	Simple mode	4	-0.153706	0.355491	0.694663	0.857524	0.427214992	1.721259443
ieu-a-1002	Leptin	ebi-a-GCST90018916	Sleep apnea syndrome	Weighted mode	4	-0.150853	0.378593	0.716958	0.859974	0.409468971	1.806133755
ieu-a-1002	Leptin	ukb-b-15622	Tuberculosis (Tb)	MR Egger	NA	NA	NA	NA	NA	NA	NA
ieu-a-1002	Leptin	ukb-b-15622	Tuberculosis (Tb)	Weighted median	NA	NA	NA	NA	NA	NA	NA
ieu-a-1002	Leptin	ukb-b-15622	Tuberculosis (Tb)	Inverse variance weighted	2	-0.000495	0.003854	0.897836	0.999505	0.991984032	1.007083615
ieu-a-1002	Leptin	ukb-b-15622	Tuberculosis (Tb)	Simple mode	NA	NA	NA	NA	NA	NA	NA
ieu-a-1002	Leptin	ukb-b-15622	Tuberculosis (Tb)	Weighted mode	NA	NA	NA	NA	NA	NA	NA

Supplementary table 4: The study used MR to estimate the relationship between leptin receptor and lung disease.

id.exposure	exposure	id.outcome	outcome	method	nsnp	b	se	pval	OR	95%CI lower	95%CI upper
prot-a-1724	Leptin receptor	finn-b-ILD	Interstitial lung disease	MR Egger	10	0.006424	0.059735	0.917011	1.006444	0.89524653	1.131454025
prot-a-1724	Leptin receptor	finn-b-ILD	Interstitial lung disease	Weighted median	10	0.010592	0.04596	0.817728	1.010649	0.9235883	1.105915435
prot-a-1724	Leptin receptor	finn-b-ILD	Interstitial lung disease	Inverse variance weighted	10	0.027419	0.041763	0.511489	1.027798	0.94701754	1.11546878
prot-a-1724	Leptin receptor	finn-b-ILD	Interstitial lung disease	Simple mode	10	0.132188	0.124007	0.314195	1.141323	0.89505919	1.455343575
prot-a-1724	Leptin receptor	finn-b-ILD	Interstitial lung disease	Weighted mode	10	0.008454	0.046728	0.860441	1.00849	0.92022918	1.105215416
prot-a-1724	Leptin receptor	finn-b-IPF	Idiopathic pulmonary fibrosis	MR Egger	10	-0.018447	0.083079	0.829843	0.981722	0.83419957	1.155332443
prot-a-1724	Leptin receptor	finn-b-IPF	Idiopathic pulmonary fibrosis	Weighted median	10	0.004343	0.063199	0.945218	1.004352	0.8873389	1.136795783
prot-a-1724	Leptin receptor	finn-b-IPF	Idiopathic pulmonary fibrosis	Inverse variance weighted	10	0.032102	0.058001	0.579946	1.032622	0.92165832	1.156946053
prot-a-1724	Leptin receptor	finn-b-IPF	Idiopathic pulmonary fibrosis	Simple mode	10	-0.054862	0.168563	0.752266	0.946616	0.68028424	1.317216804
prot-a-1724	Leptin receptor	finn-b-IPF	Idiopathic pulmonary fibrosis	Weighted mode	10	-0.001341	0.063148	0.983524	0.99866	0.8823985	1.130240004
prot-a-1724	Leptin receptor	ukb-b-18113	Asthma	MR Egger	11	0.000409	0.00102	0.697635	1.000409	0.99841052	1.00241248
prot-a-1724	Leptin receptor	ukb-b-18113	Asthma	Weighted median	11	-0.00027	0.000775	0.727714	0.99973	0.99821226	1.001250308
prot-a-1724	Leptin receptor	ukb-b-18113	Asthma	Inverse variance weighted	11	-0.000358	0.00073	0.624051	0.999642	0.99821393	1.001073039
prot-a-1724	Leptin receptor	ukb-b-18113	Asthma	Simple mode	11	-0.000383	0.002167	0.863045	0.999617	0.99538089	1.003870359
prot-a-1724	Leptin receptor	ukb-b-18113	Asthma	Weighted mode	11	-0.000267	0.000768	0.735113	0.999733	0.99822945	1.001238592
prot-a-1724	Leptin receptor	ebi-a-GCST90018918	Sarcoidosis	MR Egger	11	-0.035839	0.056247	0.539877	0.964795	0.86408526	1.077243008
prot-a-1724	Leptin receptor	ebi-a-GCST90018918	Sarcoidosis	Weighted median	11	-0.029681	0.043829	0.498272	0.970755	0.89084436	1.057833536
prot-a-1724	Leptin receptor	ebi-a-GCST90018918	Sarcoidosis	Inverse variance weighted	11	-0.02846	0.039276	0.468689	0.971941	0.89992777	1.049717395
prot-a-1724	Leptin receptor	ebi-a-GCST90018918	Sarcoidosis	Simple mode	11	-0.100742	0.104307	0.35691	0.904166	0.7369876	1.109267706
prot-a-1724	Leptin receptor	ebi-a-GCST90018918	Sarcoidosis	Weighted mode	11	-0.029675	0.043979	0.515132	0.970761	0.890588	1.058151945
prot-a-1724	Leptin receptor	ukb-b-13447	Chronic obstructive pulmonary disease	MR Egger	3	0.013026	0.022562	0.666676	1.013111	0.96928569	1.058917805
prot-a-1724	Leptin receptor	ukb-b-13447	Chronic obstructive pulmonary disease	Weighted median	3	0.000959	0.000765	0.210061	1.000959	0.9994596	1.002461257
prot-a-1724	Leptin receptor	ukb-b-13447	Chronic obstructive pulmonary disease	Inverse variance weighted	3	0.000918	0.000585	0.116414	1.000919	0.9997719	1.002066956
prot-a-1724	Leptin receptor	ukb-b-13447	Chronic obstructive pulmonary disease	Simple mode	3	0.001148	0.00083	0.300772	1.001149	0.99952138	1.002778747
prot-a-1724	Leptin receptor	ukb-b-13447	Chronic obstructive pulmonary disease	Weighted mode	3	0.001077	0.000815	0.317231	1.001078	0.99947981	1.00267807
prot-a-1724	Leptin receptor	ieu-b-4976	Pneumonia	MR Egger	11	-0.000976	0.015277	0.950475	0.999025	0.96955411	1.02939133
prot-a-1724	Leptin receptor	ieu-b-4976	Pneumonia	Weighted median	11	0.003545	0.012206	0.771505	1.003551	0.97982725	1.027849117
prot-a-1724	Leptin receptor	ieu-b-4976	Pneumonia	Inverse variance weighted	11	0.002268	0.010924	0.835566	1.00227	0.98103774	1.023962072
prot-a-1724	Leptin receptor	ieu-b-4976	Pneumonia	Simple mode	11	-0.030704	0.033568	0.381885	0.969763	0.90801354	1.035711661
prot-a-1724	Leptin receptor	ieu-b-4976	Pneumonia	Weighted mode	11	0.002127	0.011449	0.856304	1.00213	0.97989233	1.024871636
prot-a-1724	Leptin receptor	ieu-a-966	Lung cancer	MR Egger	9	0.024881	0.054722	0.663108	1.025193	0.92092656	1.141264413
prot-a-1724	Leptin receptor	ieu-a-966	Lung cancer	Weighted median	9	0.033085	0.021181	0.118278	1.033639	0.99160636	1.077452991
prot-a-1724	Leptin receptor	ieu-a-966	Lung cancer	Inverse variance weighted	9	0.036329	0.036879	0.324575	1.036997	0.96468564	1.114729022
prot-a-1724	Leptin receptor	ieu-a-966	Lung cancer	Simple mode	9	0.134735	0.112921	0.266982	1.144233	0.91705287	1.42769306
prot-a-1724	Leptin receptor	ieu-a-966	Lung cancer	Weighted mode	9	0.031974	0.022621	0.195237	1.03249	0.98771198	1.07929906
prot-a-1724	Leptin receptor	ebi-a-GCST90018916	Sleep apnea syndrome	MR Egger	11	0.027496	0.02187	0.240301	1.027878	0.98474894	1.072895528
prot-a-1724	Leptin receptor	ebi-a-GCST90018916	Sleep apnea syndrome	Weighted median	11	0.014954	0.017041	0.380202	1.015066	0.9817224	1.049543043
prot-a-1724	Leptin receptor	ebi-a-GCST90018916	Sleep apnea syndrome	Inverse variance weighted	11	0.019509	0.0154	0.205235	1.0197	0.98938099	1.050948598
prot-a-1724	Leptin receptor	ebi-a-GCST90018916	Sleep apnea syndrome	Simple mode	11	0.021968	0.045862	0.642241	1.022211	0.93433325	1.118354211
prot-a-1724	Leptin receptor	ebi-a-GCST90018916	Sleep apnea syndrome	Weighted mode	11	0.016113	0.016453	0.350506	1.016244	0.98399481	1.049549642
prot-a-1724	Leptin receptor	ukb-b-15622	Tuberculosis (Tb)	MR Egger	4	-0.000105	0.000228	0.691082	0.999895	0.99944846	1.000342258
prot-a-1724	Leptin receptor	ukb-b-15622	Tuberculosis (Tb)	Weighted median	4	-0.000218	0.000175	0.213695	0.999782	0.99943923	1.000125472
prot-a-1724	Leptin receptor	ukb-b-15622	Tuberculosis (Tb)	Inverse variance weighted	4	-0.000229	0.000173	0.185688	0.999771	0.99943308	1.000109946
prot-a-1724	Leptin receptor	ukb-b-15622	Tuberculosis (Tb)	Simple mode	4	-0.000845	0.00046	0.163546	0.999155	0.9982549	1.000056642
prot-a-1724	Leptin receptor	ukb-b-15622	Tuberculosis (Tb)	Weighted mode	4	-0.000191	0.000176	0.355274	0.999809	0.99946468	1.000152654

Supplementary table 5: The study used MR to estimate the relationship between retinol-binding protein 4 and lung disease.

id.exposure	exposure	id.outcome	outcome	method	nsnp	b	se	pval	OR	95%CI lower	95%CI upper
prot-a-2507	Retinol-binding protein 4	finn-b-ILD	Interstitial lung disease	MR Egger	14	0.063777	0.181564	0.731479	1.065855	0.74670308	1.52141628
prot-a-2507	Retinol-binding protein 4	finn-b-ILD	Interstitial lung disease	Weighted median	14	-0.046155	0.095779	0.629883	0.954894	0.79145531	1.1520832
prot-a-2507	Retinol-binding protein 4	finn-b-ILD	Interstitial lung disease	Inverse variance weighted	14	-0.066458	0.071608	0.353361	0.935702	0.813175	1.07669118
prot-a-2507	Retinol-binding protein 4	finn-b-ILD	Interstitial lung disease	Simple mode	14	-0.058548	0.153717	0.709444	0.943133	0.69779331	1.27473374
prot-a-2507	Retinol-binding protein 4	finn-b-ILD	Interstitial lung disease	Weighted mode	14	-0.056178	0.151834	0.717344	0.945371	0.7020342	1.27305206
prot-a-2507	Retinol-binding protein 4	finn-b-IPF	Idiopathic pulmonary fibrosis	MR Egger	14	0.084775	0.253679	0.744012	1.088472	0.66203494	1.78958909
prot-a-2507	Retinol-binding protein 4	finn-b-IPF	Idiopathic pulmonary fibrosis	Weighted median	14	-0.070571	0.134939	0.600985	0.931861	0.71530114	1.2139864
prot-a-2507	Retinol-binding protein 4	finn-b-IPF	Idiopathic pulmonary fibrosis	Inverse variance weighted	14	-0.162806	0.099611	0.102173	0.849756	0.69904215	1.0329641
prot-a-2507	Retinol-binding protein 4	finn-b-IPF	Idiopathic pulmonary fibrosis	Simple mode	14	-0.128069	0.196068	0.52502	0.879793	0.59907835	1.29204286
prot-a-2507	Retinol-binding protein 4	finn-b-IPF	Idiopathic pulmonary fibrosis	Weighted mode	14	-0.084276	0.194588	0.672042	0.919178	0.62771537	1.34597216
prot-a-2507	Retinol-binding protein 4	ukb-b-18113	Asthma	MR Egger	12	0.005134	0.008827	0.573681	1.005148	0.9879065	1.02268964
prot-a-2507	Retinol-binding protein 4	ukb-b-18113	Asthma	Weighted median	12	0.000781	0.002453	0.750106	1.000782	0.99598128	1.00560511
prot-a-2507	Retinol-binding protein 4	ukb-b-18113	Asthma	Inverse variance weighted	12	0.004904	0.003082	0.111608	1.004916	0.99886332	1.01100513
prot-a-2507	Retinol-binding protein 4	ukb-b-18113	Asthma	Simple mode	12	-0.000622	0.003367	0.856853	0.999378	0.9928049	1.00599548
prot-a-2507	Retinol-binding protein 4	ukb-b-18113	Asthma	Weighted mode	12	-0.000356	0.003749	0.926009	0.999644	0.99232609	1.00701562
prot-a-2507	Retinol-binding protein 4	ebi-a-GCST90018918	Sarcoidosis	MR Egger	14	-0.188909	0.304214	0.546231	0.827862	0.4560426	1.50283144
prot-a-2507	Retinol-binding protein 4	ebi-a-GCST90018918	Sarcoidosis	Weighted median	14	-0.076099	0.110027	0.489164	0.926725	0.74695439	1.14976054
prot-a-2507	Retinol-binding protein 4	ebi-a-GCST90018918	Sarcoidosis	Inverse variance weighted	14	-0.081894	0.107603	0.446612	0.92137	0.74617489	1.13769882
prot-a-2507	Retinol-binding protein 4	ebi-a-GCST90018918	Sarcoidosis	Simple mode	14	-0.074374	0.169757	0.66849	0.928324	0.66557861	1.29479189
prot-a-2507	Retinol-binding protein 4	ebi-a-GCST90018918	Sarcoidosis	Weighted mode	14	-0.074374	0.161057	0.651865	0.928324	0.67702599	1.2728991
prot-a-2507	Retinol-binding protein 4	ukb-b-13447	Chronic obstructive pulmonary disease	MR Egger	3	-0.007668	0.004099	0.312519	0.992362	0.98442162	1.00036593
prot-a-2507	Retinol-binding protein 4	ukb-b-13447	Chronic obstructive pulmonary disease	Weighted median	3	-0.000351	0.000699	0.615205	0.999649	0.99828085	1.00101875
prot-a-2507	Retinol-binding protein 4	ukb-b-13447	Chronic obstructive pulmonary disease	Inverse variance weighted	3	1.04E-06	0.000785	0.998942	1.000001	0.99846278	1.00154167
prot-a-2507	Retinol-binding protein 4	ukb-b-13447	Chronic obstructive pulmonary disease	Simple mode	3	-0.000452	0.000987	0.692117	0.999548	0.99761574	1.00148448
prot-a-2507	Retinol-binding protein 4	ukb-b-13447	Chronic obstructive pulmonary disease	Weighted mode	3	-0.000626	0.000792	0.512075	0.999374	0.99782507	1.00092619
prot-a-2507	Retinol-binding protein 4	ieu-b-4976	Pneumonia	MR Egger	11	0.10518	0.069737	0.165768	1.110911	0.96898638	1.27362187
prot-a-2507	Retinol-binding protein 4	ieu-b-4976	Pneumonia	Weighted median	11	-0.019761	0.032885	0.547896	0.980433	0.91923408	1.04570662
prot-a-2507	Retinol-binding protein 4	ieu-b-4976	Pneumonia	Inverse variance weighted	11	0.017678	0.024879	0.477367	1.017835	0.96939296	1.06869732
prot-a-2507	Retinol-binding protein 4	ieu-b-4976	Pneumonia	Simple mode	11	-0.024352	0.050883	0.642525	0.975942	0.88330757	1.07829204
prot-a-2507	Retinol-binding protein 4	ieu-b-4976	Pneumonia	Weighted mode	11	-0.0238	0.0506	0.648188	0.976481	0.88428565	1.07828843
prot-a-2507	Retinol-binding protein 4	ieu-a-966	Lung cancer	MR Egger	9	0.16249	0.217296	0.478955	1.176437	0.76842634	1.8010873
prot-a-2507	Retinol-binding protein 4	ieu-a-966	Lung cancer	Weighted median	9	0.047493	0.070458	0.500274	1.048639	0.91337866	1.20392871
prot-a-2507	Retinol-binding protein 4	ieu-a-966	Lung cancer	Inverse variance weighted	9	0.072487	0.050678	0.152613	1.075179	0.9735163	1.18745867
prot-a-2507	Retinol-binding protein 4	ieu-a-966	Lung cancer	Simple mode	9	0.046622	0.11023	0.683468	1.047726	0.8441473	1.30040105
prot-a-2507	Retinol-binding protein 4	ieu-a-966	Lung cancer	Weighted mode	9	0.045334	0.114524	0.702562	1.046378	0.83599539	1.30970398
prot-a-2507	Retinol-binding protein 4	ebi-a-GCST90018916	Sleep apnea syndrome	MR Egger	14	-0.066245	0.079943	0.42348	0.935902	0.8001677	1.09466049
prot-a-2507	Retinol-binding protein 4	ebi-a-GCST90018916	Sleep apnea syndrome	Weighted median	14	-0.02898	0.039184	0.45955	0.971436	0.89962125	1.04898288
prot-a-2507	Retinol-binding protein 4	ebi-a-GCST90018916	Sleep apnea syndrome	Inverse variance weighted	14	-0.03874	0.029522	0.189436	0.962001	0.90791731	1.01930666
prot-a-2507	Retinol-binding protein 4	ebi-a-GCST90018916	Sleep apnea syndrome	Simple mode	14	-0.022673	0.070732	0.753643	0.977582	0.85102966	1.12295263
prot-a-2507	Retinol-binding protein 4	ebi-a-GCST90018916	Sleep apnea syndrome	Weighted mode	14	-0.012182	0.070425	0.86533	0.987892	0.86052361	1.13411191
prot-a-2507	Retinol-binding protein 4	ukb-b-15622	Tuberculosis (Tb)	MR Egger	5	-0.002192	0.004593	0.665769	0.99781	0.98886872	1.00683283
prot-a-2507	Retinol-binding protein 4	ukb-b-15622	Tuberculosis (Tb)	Weighted median	5	-0.000267	0.000649	0.68097	0.999733	0.99846132	1.00100637
prot-a-2507	Retinol-binding protein 4	ukb-b-15622	Tuberculosis (Tb)	Inverse variance weighted	5	-0.000202	0.000524	0.700103	0.999798	0.99877247	1.00082515
prot-a-2507	Retinol-binding protein 4	ukb-b-15622	Tuberculosis (Tb)	Simple mode	5	-7.98E-05	0.000843	0.929086	0.99992	0.99826951	1.00157353
prot-a-2507	Retinol-binding protein 4	ukb-b-15622	Tuberculosis (Tb)	Weighted mode	5	-0.00031	0.000846	0.732889	0.99969	0.99803309	1.00135013

Supplementary table 6: The study used MR to estimate the relationship between resistin and lung disease.

id.exposure	exposure	id.outcome	outcome	method	nsnp	b	se	pval	OR	95%CI lower	95%CI upper
prot-a-2524	Resistin	finn-b-ILD	Interstitial lung disease	MR Egger	14	0.063524	0.212824	0.77044	1.065585	0.70214793	1.617141019
prot-a-2524	Resistin	finn-b-ILD	Interstitial lung disease	Weighted median	14	0.067018	0.106997	0.531078	1.069315	0.86701867	1.318812401
prot-a-2524	Resistin	finn-b-ILD	Interstitial lung disease	Inverse variance weighted	14	0.005231	0.093044	0.955165	1.005245	0.83766674	1.206347564
prot-a-2524	Resistin	finn-b-ILD	Interstitial lung disease	Simple mode	14	0.133112	0.201584	0.520573	1.142378	0.76951605	1.695905492
prot-a-2524	Resistin	finn-b-ILD	Interstitial lung disease	Weighted mode	14	0.115844	0.170696	0.509257	1.122821	0.8035466	1.56895266
prot-a-2524	Resistin	finn-b-IPF	Idiopathic pulmonary fibrosis	MR Egger	14	-0.020486	0.22413	0.928682	0.979723	0.63142283	1.520149124
prot-a-2524	Resistin	finn-b-IPF	Idiopathic pulmonary fibrosis	Weighted median	14	-0.006062	0.131556	0.96325	0.993957	0.76804122	1.286324262
prot-a-2524	Resistin	finn-b-IPF	Idiopathic pulmonary fibrosis	Inverse variance weighted	14	0.009243	0.101359	0.927338	1.009286	0.82743819	1.231099405
prot-a-2524	Resistin	finn-b-IPF	Idiopathic pulmonary fibrosis	Simple mode	14	-0.073754	0.210107	0.73119	0.928901	0.61535037	1.402219366
prot-a-2524	Resistin	finn-b-IPF	Idiopathic pulmonary fibrosis	Weighted mode	14	-0.034377	0.182625	0.853597	0.966207	0.67548675	1.382049927
prot-a-2524	Resistin	ukb-b-18113	Asthma	MR Egger	14	-0.003114	0.007532	0.686542	0.996891	0.98228225	1.011716084
prot-a-2524	Resistin	ukb-b-18113	Asthma	Weighted median	14	-0.002017	0.001888	0.285404	0.997985	0.99429914	1.001684977
prot-a-2524	Resistin	ukb-b-18113	Asthma	Inverse variance weighted	14	0.00136	0.003511	0.69856	1.00136	0.99449394	1.0082744
prot-a-2524	Resistin	ukb-b-18113	Asthma	Simple mode	14	-0.002203	0.002771	0.4409	0.9978	0.99239608	1.003233045
prot-a-2524	Resistin	ukb-b-18113	Asthma	Weighted mode	14	-0.002666	0.002356	0.278292	0.997338	0.99274349	1.001953732
prot-a-2524	Resistin	ebi-a-GCST90018918	Sarcoidosis	MR Egger	15	-0.052988	0.145358	0.721319	0.948391	0.71327347	1.261011146
prot-a-2524	Resistin	ebi-a-GCST90018918	Sarcoidosis	Weighted median	15	0.053512	0.094045	0.569352	1.05497	0.87737975	1.268505328
prot-a-2524	Resistin	ebi-a-GCST90018918	Sarcoidosis	Inverse variance weighted	15	-0.011297	0.06887	0.869705	0.988767	0.86391442	1.131662508
prot-a-2524	Resistin	ebi-a-GCST90018918	Sarcoidosis	Simple mode	15	0.101762	0.162748	0.54185	1.10712	0.80474955	1.523100457
prot-a-2524	Resistin	ebi-a-GCST90018918	Sarcoidosis	Weighted mode	15	0.105179	0.145345	0.481198	1.110909	0.83552351	1.477061028
prot-a-2524	Resistin	ukb-b-13447	Chronic obstructive pulmonary disease	MR Egger	4	0.009807	0.006689	0.280276	1.009855	0.99670161	1.023181974
prot-a-2524	Resistin	ukb-b-13447	Chronic obstructive pulmonary disease	Weighted median	4	-0.000841	0.000645	0.192046	0.999159	0.99789696	1.000422777
prot-a-2524	Resistin	ukb-b-13447	Chronic obstructive pulmonary disease	Inverse variance weighted	4	-0.000751	0.000494	0.128151	0.999249	0.99828264	1.00021655
prot-a-2524	Resistin	ukb-b-13447	Chronic obstructive pulmonary disease	Simple mode	4	-0.000978	0.00095	0.379231	0.999023	0.99716388	1.000885173
prot-a-2524	Resistin	ukb-b-13447	Chronic obstructive pulmonary disease	Weighted mode	4	-0.000914	0.000836	0.354489	0.999087	0.99745033	1.000725772
prot-a-2524	Resistin	ieu-b-4976	Pneumonia	MR Egger	16	0.011285	0.047686	0.81636	1.011349	0.92110548	1.110433231
prot-a-2524	Resistin	ieu-b-4976	Pneumonia	Weighted median	16	-0.041671	0.026824	0.120302	0.959186	0.91005968	1.01096332
prot-a-2524	Resistin	ieu-b-4976	Pneumonia	Inverse variance weighted	16	0.001781	0.022027	0.935562	1.001782	0.95945301	1.045979377
prot-a-2524	Resistin	ieu-b-4976	Pneumonia	Simple mode	16	-0.059463	0.058929	0.328943	0.94227	0.83948752	1.057637044
prot-a-2524	Resistin	ieu-b-4976	Pneumonia	Weighted mode	16	-0.048233	0.040408	0.251158	0.952912	0.88035265	1.031451893
prot-a-2524	Resistin	ieu-a-966	Lung cancer	MR Egger	11	0.09272	0.11414	0.437559	1.097155	0.8772221	1.372227301
prot-a-2524	Resistin	ieu-a-966	Lung cancer	Weighted median	11	0.01999	0.055674	0.719563	1.020191	0.91472441	1.137817084
prot-a-2524	Resistin	ieu-a-966	Lung cancer	Inverse variance weighted	11	0.002293	0.042391	0.956861	1.002296	0.92238468	1.089129777
prot-a-2524	Resistin	ieu-a-966	Lung cancer	Simple mode	11	0.032889	0.089256	0.720192	1.033436	0.86757663	1.231004371
prot-a-2524	Resistin	ieu-a-966	Lung cancer	Weighted mode	11	0.017339	0.085229	0.842868	1.017491	0.86095792	1.202482759
prot-a-2524	Resistin	ebi-a-GCST90018916	Sleep apnea syndrome	MR Egger	15	-0.024695	0.065826	0.713593	0.975607	0.85751762	1.109959469
prot-a-2524	Resistin	ebi-a-GCST90018916	Sleep apnea syndrome	Weighted median	15	-0.074662	0.038685	0.053602	0.928057	0.86029141	1.001159835
prot-a-2524	Resistin	ebi-a-GCST90018916	Sleep apnea syndrome	Inverse variance weighted	15	-0.056958	0.030494	0.061787	0.944634	0.88982834	1.002814931
prot-a-2524	Resistin	ebi-a-GCST90018916	Sleep apnea syndrome	Simple mode	15	-0.086606	0.058232	0.159119	0.917039	0.8181252	1.027910743
prot-a-2524	Resistin	ebi-a-GCST90018916	Sleep apnea syndrome	Weighted mode	15	-0.086606	0.04834	0.094834	0.917039	0.83414083	1.008174707
prot-a-2524	Resistin	ukb-b-15622	Tuberculosis (Tb)	MR Egger	4	0.009559	0.007966	0.352996	1.009605	0.99396424	1.025492548
prot-a-2524	Resistin	ukb-b-15622	Tuberculosis (Tb)	Weighted median	4	-5.31E-06	0.000679	0.993755	0.999995	0.9986648	1.001326344
prot-a-2524	Resistin	ukb-b-15622	Tuberculosis (Tb)	Inverse variance weighted	4	0.000217	0.000588	0.712397	1.000217	0.99906476	1.001370109
prot-a-2524	Resistin	ukb-b-15622	Tuberculosis (Tb)	Simple mode	4	-0.000243	0.000978	0.819681	0.999757	0.99784207	1.001675252
prot-a-2524	Resistin	ukb-b-15622	Tuberculosis (Tb)	Weighted mode	4	-0.000235	0.000961	0.822277	0.999765	0.99788297	1.001649725

Supplementary table 7: The study used MR to estimate the relationship between plasminogen activator inhibitor 1 and lung disease.

id.exposure	exposure	id.outcome	outcome	method	nsnp	b	se	pval	OR	95%CI lower	95%CI upper
prot-a-2696	Plasminogen activator inhibitor 1	finn-b-ILD	Interstitial lung disease	MR Egger	17	-0.031618	0.183192	0.865279	0.968877	0.67660021	1.38741131
prot-a-2696	Plasminogen activator inhibitor 1	finn-b-ILD	Interstitial lung disease	Weighted median	17	-0.013752	0.09211	0.881316	0.986342	0.82342142	1.18149773
prot-a-2696	Plasminogen activator inhibitor 1	finn-b-ILD	Interstitial lung disease	Inverse variance weighted	17	-0.025441	0.067029	0.704276	0.97488	0.85486022	1.11174986
prot-a-2696	Plasminogen activator inhibitor 1	finn-b-ILD	Interstitial lung disease	Simple mode	17	-0.005179	0.16221	0.974924	0.994834	0.72389305	1.3671845
prot-a-2696	Plasminogen activator inhibitor 1	finn-b-ILD	Interstitial lung disease	Weighted mode	17	-0.002611	0.149569	0.986289	0.997393	0.74396143	1.33715549
prot-a-2696	Plasminogen activator inhibitor 1	finn-b-IPF	Idiopathic pulmonary fibrosis	MR Egger	17	-0.148363	0.253621	0.567258	0.862118	0.52442163	1.41727137
prot-a-2696	Plasminogen activator inhibitor 1	finn-b-IPF	Idiopathic pulmonary fibrosis	Weighted median	17	-0.084391	0.121666	0.487916	0.919072	0.72407846	1.16657735
prot-a-2696	Plasminogen activator inhibitor 1	finn-b-IPF	Idiopathic pulmonary fibrosis	Inverse variance weighted	17	-0.113003	0.092907	0.223869	0.893148	0.74445613	1.07153745
prot-a-2696	Plasminogen activator inhibitor 1	finn-b-IPF	Idiopathic pulmonary fibrosis	Simple mode	17	-0.068683	0.192883	0.726427	0.933623	0.63971422	1.36256315
prot-a-2696	Plasminogen activator inhibitor 1	finn-b-IPF	Idiopathic pulmonary fibrosis	Weighted mode	17	-0.075346	0.211789	0.726666	0.927422	0.61234934	1.40461017
prot-a-2696	Plasminogen activator inhibitor 1	ukb-b-18113	Asthma	MR Egger	17	0.00573	0.004789	0.249999	1.005747	0.99635156	1.01523072
prot-a-2696	Plasminogen activator inhibitor 1	ukb-b-18113	Asthma	Weighted median	17	0.000468	0.001994	0.814401	1.000468	0.99656498	1.00438694
prot-a-2696	Plasminogen activator inhibitor 1	ukb-b-18113	Asthma	Inverse variance weighted	17	-0.001027	0.001796	0.56743	0.998974	0.99546347	1.00249606
prot-a-2696	Plasminogen activator inhibitor 1	ukb-b-18113	Asthma	Simple mode	17	0.000768	0.003375	0.822949	1.000768	0.99417046	1.00740906
prot-a-2696	Plasminogen activator inhibitor 1	ukb-b-18113	Asthma	Weighted mode	17	0.00114	0.002875	0.697017	1.00114	0.99551468	1.00679803
prot-a-2696	Plasminogen activator inhibitor 1	ebi-a-GCST90018918	Sarcoidosis	MR Egger	17	-0.094728	0.199366	0.641525	0.90962	0.61539855	1.3445081
prot-a-2696	Plasminogen activator inhibitor 1	ebi-a-GCST90018918	Sarcoidosis	Weighted median	17	0.019299	0.093631	0.836697	1.019487	0.84855709	1.22484763
prot-a-2696	Plasminogen activator inhibitor 1	ebi-a-GCST90018918	Sarcoidosis	Inverse variance weighted	17	0.023199	0.069222	0.737519	1.02347	0.89361912	1.17218993
prot-a-2696	Plasminogen activator inhibitor 1	ebi-a-GCST90018918	Sarcoidosis	Simple mode	17	0.027955	0.186656	0.882819	1.02835	0.71327292	1.48260638
prot-a-2696	Plasminogen activator inhibitor 1	ebi-a-GCST90018918	Sarcoidosis	Weighted mode	17	0.031637	0.178222	0.861331	1.032143	0.7278375	1.46367704
prot-a-2696	Plasminogen activator inhibitor 1	ukb-b-13447	Chronic obstructive pulmonary disease	MR Egger	4	-0.001292	0.001932	0.572464	0.998709	0.99493499	1.00249713
prot-a-2696	Plasminogen activator inhibitor 1	ukb-b-13447	Chronic obstructive pulmonary disease	Weighted median	4	0.000885	0.000544	0.104182	1.000885	0.99981761	1.00195331
prot-a-2696	Plasminogen activator inhibitor 1	ukb-b-13447	Chronic obstructive pulmonary disease	Inverse variance weighted	4	0.000809	0.000445	0.069092	1.000809	0.99993673	1.00168263
prot-a-2696	Plasminogen activator inhibitor 1	ukb-b-13447	Chronic obstructive pulmonary disease	Simple mode	4	0.001166	0.000782	0.232677	1.001167	0.99963354	1.00270274
prot-a-2696	Plasminogen activator inhibitor 1	ukb-b-13447	Chronic obstructive pulmonary disease	Weighted mode	4	0.000265	0.0006	0.688846	1.000265	0.99908868	1.00144268
prot-a-2696	Plasminogen activator inhibitor 1	ieu-b-4976	Pneumonia	MR Egger	15	0.105253	0.055703	0.081327	1.110992	0.99608283	1.23915748
prot-a-2696	Plasminogen activator inhibitor 1	ieu-b-4976	Pneumonia	Weighted median	15	0.024531	0.029862	0.411375	1.024835	0.96657232	1.08660892
prot-a-2696	Plasminogen activator inhibitor 1	ieu-b-4976	Pneumonia	Inverse variance weighted	15	0.026727	0.021245	0.208376	1.027087	0.98519779	1.07075753
prot-a-2696	Plasminogen activator inhibitor 1	ieu-b-4976	Pneumonia	Simple mode	15	0.021016	0.051446	0.689081	1.021238	0.92328463	1.12958433
prot-a-2696	Plasminogen activator inhibitor 1	ieu-b-4976	Pneumonia	Weighted mode	15	0.024305	0.046012	0.605615	1.024603	0.93624402	1.1213002
prot-a-2696	Plasminogen activator inhibitor 1	ieu-a-966	Lung cancer	MR Egger	15	0.016126	0.112873	0.888582	1.016257	0.8145615	1.26789514
prot-a-2696	Plasminogen activator inhibitor 1	ieu-a-966	Lung cancer	Weighted median	15	0.035343	0.053114	0.505786	1.035975	0.93354953	1.14963833
prot-a-2696	Plasminogen activator inhibitor 1	ieu-a-966	Lung cancer	Inverse variance weighted	15	0.002746	0.039953	0.945194	1.00275	0.92722328	1.08442928
prot-a-2696	Plasminogen activator inhibitor 1	ieu-a-966	Lung cancer	Simple mode	15	0.039335	0.087531	0.660035	1.040119	0.87614291	1.2347835
prot-a-2696	Plasminogen activator inhibitor 1	ieu-a-966	Lung cancer	Weighted mode	15	0.050998	0.076061	0.513457	1.052321	0.90657495	1.22149707
prot-a-2696	Plasminogen activator inhibitor 1	ebi-a-GCST90018916	Sleep apnea syndrome	MR Egger	17	-0.138747	0.081223	0.108199	0.870448	0.74234346	1.02065997
prot-a-2696	Plasminogen activator inhibitor 1	ebi-a-GCST90018916	Sleep apnea syndrome	Weighted median	17	-0.046961	0.035428	0.18499	0.954125	0.89011964	1.02273169
prot-a-2696	Plasminogen activator inhibitor 1	ebi-a-GCST90018916	Sleep apnea syndrome	Inverse variance weighted	17	-0.023058	0.030482	0.449393	0.977206	0.92053204	1.03736918
prot-a-2696	Plasminogen activator inhibitor 1	ebi-a-GCST90018916	Sleep apnea syndrome	Simple mode	17	-0.066619	0.063547	0.310057	0.935552	0.82599202	1.05964322
prot-a-2696	Plasminogen activator inhibitor 1	ebi-a-GCST90018916	Sleep apnea syndrome	Weighted mode	17	-0.05625	0.057743	0.344484	0.945303	0.84414926	1.05857776
prot-a-2696	Plasminogen activator inhibitor 1	ukb-b-15622	Tuberculosis (Tb)	MR Egger	6	0.002216	0.002299	0.389649	1.002218	0.99771304	1.00674398
prot-a-2696	Plasminogen activator inhibitor 1	ukb-b-15622	Tuberculosis (Tb)	Weighted median	6	0.000258	0.000586	0.659766	1.000258	0.9991091	1.00140866
prot-a-2696	Plasminogen activator inhibitor 1	ukb-b-15622	Tuberculosis (Tb)	Inverse variance weighted	6	-0.000195	0.000459	0.67105	0.999805	0.9989053	1.00070541
prot-a-2696	Plasminogen activator inhibitor 1	ukb-b-15622	Tuberculosis (Tb)	Simple mode	6	0.000318	0.00102	0.767921	1.000318	0.99832041	1.00231921
prot-a-2696	Plasminogen activator inhibitor 1	ukb-b-15622	Tuberculosis (Tb)	Weighted mode	6	0.0005	0.000757	0.53817	1.0005	0.99901714	1.00198482
